# Reuse Distance-Aided Resource Selection Mechanisms for NR-V2X Sidelink Communication

**DOI:** 10.3390/s24010253

**Published:** 2023-12-31

**Authors:** Jicheng Yin, Seung-Hoon Hwang

**Affiliations:** Division of Electronics and Electrical Engineering, Dongguk University-Seoul, Seoul 04620, Republic of Korea; jicheng.yin@dgu.ac.kr

**Keywords:** cellular vehicle-to-everything (C-V2X), NR-V2X, sidelink, Mode 2, SB-SPS, resource selection

## Abstract

Cellular vehicle-to-everything (C-V2X) facilitates direct communication between vehicles and other user equipment (UE) to improve the efficiency of the Internet of vehicles communication through sidelink. In addition, in the new radio vehicle-to-everything (NR-V2X) Mode 2, users can automatically select resources using the conventional sensing-based semi-persistent scheduling (SB-SPS) resource selection algorithm. This mechanism allows users to generate a list of available resources after a sensing window, after which the users can randomly select resources, and the resource can be used continuously over multiple periods before reselection. However, during the sensing window, neighbors may generate a similar list of available resources, and random selection may lead to resource conflicts. This phenomenon may lead to deteriorated communication performance and increased latency due to incorrect reception. Therefore, this paper proposes a reuse distance-aided resource selection (RD-RS) method which integrates resource reuse distance judgement with SB-SPS to mitigate resource conflicts and interference caused by random selection. Moreover, the reuse distance judgement is performed before the final resource selection, and whether the user will select the current resource depends on the reuse distance between that user and other occupiers. Furthermore, the performance of the proposed scheme is compared with other algorithms. Simulation results show that the proposed RD-RS not only achieves a higher packet reception ratio (PRR) but also effectively reduces the inter-packet gap (IPG). Moreover, in specific scenarios, the proposed method outperforms conventional schemes by 9% in terms of PRR and 70% in terms of Range.

## 1. Introduction

Vehicle-to-everything (V2X) is an advanced technology that enables all-round connectivity and communication between vehicles and vulnerable road users. C-V2X extends V2X by integrating cellular techniques to facilitate direct communication between terminals, encompassing LTE-V2X and NR-V2X. C-V2X can support the establishment of communication links between vehicles and various user equipment (UE), including vehicle-to-vehicle (V2V), vehicle-to-pedestrian (V2P), vehicle-to-infrastructure (V2I), and vehicle-to-network (V2N) communication [1]. With the connection between the vehicle and the network, the vehicle can obtain self-information, such as position and speed, through the global navigation satellite system (GNSS) and sensor devices. Additionally, because all users are connected, the surrounding status can be obtained by communicating with neighbors. This information can be incorporated in cooperative awareness messages (CAMs) as a periodic message. Based on the obtained information, a vehicle can plan its own driving path planning and take appropriate actions in emergency situations.

The initial standard supporting LTE-V2X services was completed in Release 14 [2], primarily aimed at satisfying the requirements of essential road safety services. Further enhancements, focusing on additional V2X operation scenarios, were completed in Release 15 [3]. The technical specifications for NR-V2X were introduced in Release 16 [4]. In addition to enhancing the interface, this standard identified four groups of use cases for advanced V2X services: vehicle platooning, extended sensors, advanced driving, and remote driving [5]. Moreover, C-V2X includes two resource allocation schemes: centralized mode (Mode 3 in LTE-V2X and Mode 1 in NR-V2X) and distributed mode (Mode 4 in LTE-V2X and Mode 2 in NR-V2X). In the centralized mode, the base station (BS) manages and allocates resources, allowing UEs to communicate through the uplink (UL) and downlink (DL) with the Uu interface. In contrast, in the distributed mode, UEs can independently select resources and communicate through the sidelink (SL) with the PC5 interface [6]. Distributed resource selection is based on sensing information rather than relying solely on the BS to meet service requirements. Specifically, in the NR-V2X Mode 2, SB-SPS is implemented for resource selection, allowing UEs to randomly choose a resource from the available pool, with the option to reserve the same resource for several periods.

**Figure 1:**
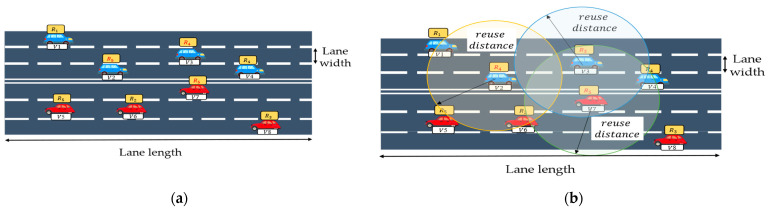
Resource selection: (**a**) random selection and (**b**) selection with reuse distance judgement.

Although certain resources may be excluded from the sensing window in SB-SPS, adjacent users are likely to generate similar lists of available resources during this window, potentially resulting in the selection of the same resource. Several previous studies have recognized the importance of resource allocation algorithms (RAM). However, most of the existing schemes focus only on adjusting specific parameter values, such as the sensing duration or reselection probability, rather than improving the resource selection process. Additionally, these frameworks rely on random resource selection, which may lead to resource conflicts, especially in high-density scenarios. Interference from other UEs sharing the same resources also affects the determination of the signal-to-interference-plus-noise ratio (SINR). Therefore, resource conflict represents a significant problem in the distributed resource selection mode. However, the conventional mechanism does not specify the conditions that UEs should satisfy when selecting reusable resources. In the case shown in Figure 1a, V2, V3, and V7 require resource selection. Owing to similar situations, there may be significant overlap in the list of available resources. Moreover, because of random selection, three vehicles may choose the same resource or a resource with high interference, resulting in poor performance. To solve the problems, resource selection algorithms in Mode 2 must be enhanced to improve the resource allocation efficiency. To this end, an effective approach may be to implement reuse distance judgement before resource selection. Here, the reuse distance is the minimum distance allowing different transmitters to use the same resource, and its accurate determination can reduce the likelihood of resource conflict among neighbors. As shown in Figure 1b, the introduction of reuse distance constraints can prevent adjacent users from using the same resources. Considering this aspect, the paper proposes an enhanced algorithm for resource selection integrating reuse distance judgement, named reuse distance-aided resource selection (RD-RS). RD-RS retains the structure of the conventional SB-SPS, while incorporating the evaluation of the reuse distance for each candidate resource.

Overall, the key contributions of this work can be summarized as follows.

- The proposed approach involves sorting available resources in ascending order based on the received power value. Subsequently, each resource is sequentially evaluated based on the reuse distance. This scheme can reduce the possibility of neighboring users using the same resource and minimize resource interference.
- The performance of the proposed RD-RS and conventional SB-SPS is theoretically analyzed under different scenarios. In terms of the resource reuse distance, we consider not only a fixed value but also an adaptive value based on the scenario setting.
- Comprehensive evaluations are performed using the packet reception ratio (PRR), error block rate, range, and inter-packet gap (IPG). Simulation and numerical results verify the effectiveness of the proposed scheme over conventional schemes in various scenarios.

The remaining article is organized as follows. Section 2 describes the related works, and Section 3 introduces the relevant technologies. The proposed scheme is outlined in Section 4. The performance evaluation is described in Section 5. Section 6 presents the concluding remarks.

## 2. Related Works

Recent studies have comprehensively explored the characteristics of C-V2X. Several researchers extensively studied the performance analysis and comparison of various technologies to identify the approaches that can meet the service quality requirements for vehicular applications. Additionally, resource allocation and improvement of parameter adaptation schemes in V2X have emerged as research hotspots, with several researchers exploring the integration of artificial intelligence (AI) algorithms.

In addition to C-V2X, IEEE 802.11p can support short-range communication between vehicles. In particular, IEEE 802.11p, the first wireless standard for vehicle networking, was released as an official standard in 2010 and enables communication between vehicles and roadside units. The evolution of this technology is IEEE 802.11bd, the standardization of which began in 2018. IEEE 802.11bd serves as the foundation for dedicated short-range communication (DSRC) in the US and intelligent transport systems (ITS-G5) in Europe. The reliability and performance analysis of upcoming vehicular communication technologies (i.e., IEEE 802.11bd and NR-V2X) have been discussed in [7], in which the authors outlined the objectives of these technologies, corresponding features, and key mechanisms. The physical layer performance of several V2X communication technologies, including LTE-V2X, NR-V2X, and DSRC, have been evaluated and compared to identify the technology most suitable for V2X communication in [8]. NR-V2X was noted to outperform all other technologies in terms of reliability, range, latency, and data rate. The progress of NR-V2X technology has been evaluated through a comparative analysis with LTE-V2X in [9]. Tutorials on NR-V2X technology standardization, architecture, resource selection, and challenges have also been presented in [10,11,12], highlighting the characteristics of NR-V2X technology, application scenarios, technical framework, and related studies. Despite extensive research efforts in recent years, the role of NR-V2X in future connected vehicle communication networks remains unclear. To fill this gap and stimulate future research, the design considerations, technological elements, capabilities, and key attributes of NR-V2X for cooperative autonomous driving were systematically highlighted in [13]. Additionally, focusing on the channel aspect, a fundamental analysis of NR-V2X was presented in [14], and their work primarily provides performance evaluations to assess the benefits of novel control channel designs.

Additionally, the body of research on SB-SPS resource selection algorithms has rapidly grown. Certain researchers evaluated the performance of SB-SPS [15,16,17,18,19], while others performed system-level simulations of NR-V2X Mode 2 parameters, including numerology, selection window size, and keep probability [20]. Meanwhile, the basic performance analysis of NR-V2X has been presented in [21]. These studies demonstrated the effectiveness of open-source simulators and clarified the impact of NR-V2X on physical layer parameters and the RAM performance. In addition, the C-V2X RAM was comprehensively discussed in [22,23]. These works described and compared the RAM in different modes, while offering potential solutions for future challenges that the resource allocation algorithm should address. The authors highlighted that RAM should effectively allocate resources to users to ensure the quality of service. In addition, several new algorithms have been established to enhance the resource allocation effectiveness of SB-SPS. This is important as the random selection inherent in the distributed SB-SPS algorithm may result in resource conflicts when using the C-V2X distributed mode, especially in congested scenarios [24]. For a given setting, a growing number of users will inevitably lead to an increase in conflicts over resource selection. To reduce the error reception due to resource conflicts, a short-term sensing-based resource selection mechanism was proposed in [25], where a short-term sensing duration is configured at the beginning of the resource unit right before resource selection, and whether the packet is ultimately transmitted on the selected resource depends on the sensing result. Similarly, to avoid resource conflicts, a resource pre-emption mechanism considering priority was proposed in [26], and a two-stage resource management mechanism based on vehicle density was designed in [27]. Additionally, an automatic resource selection mechanism was proposed in [28] based on the heading direction to minimize potential conflicts among neighboring resources. To adjust the maintenance period of the resource adaptively according to the state of each user, an algorithm for adaptively modifying the reselection probability according to the user channel status information (CSI) was proposed in [19].

Moreover, an increasing number of studies have integrated AI algorithms within the domain of wireless communications. Given the key role of resource allocation in V2X, efforts have been made to enhance communication efficiency through the application of deep learning and machine learning, as outlined in [29] and [30], respectively. A two-phase fuzzy logic-based handoff scheme was proposed in [31], demonstrating its ability to reduce unnecessary handoffs and decision delays compared with conventional schemes. Also, the combination of deep reinforcement learning (RL) with resource allocation has been explored in [32,33,34,35]. Through the RL mechanism, parameter values and resource allocation are optimized based on the information learned from the environment, thereby enhancing communication performance.

## 3. Overview of NR-V2X

This section first describes the resource configuration and physical layer in NR-V2X. Subsequently, the SB-SPS resource allocation algorithm adopted in distributed NR-V2X Mode 2 will be discussed.

### 3.1. NR-V2X Time–Frequency Resource and Physical Layer Configuration

Orthogonal resources in NR-V2X are two-dimensional structures spanning both the time and frequency domains. In the time domain, the signal is represented by a frame, and the transmission time parameters of the data are constrained by the frame structure to ensure the correct operation of the transmitter and receiver. The duration of a frame is $T_{f}$ = 10 ms, including 10 subframes, each with a duration of $T_{sf}$ = 1 ms. Each subframe further includes several slots, and the number of slots is directly related to the subcarrier spacing (SCS) configuration. Notably, the SCS size in NR-V2X 15, 30, 60, and 120 kHz, unlike the LTE-V2X, which supports only 15 kHz. A larger SCS results in a shorter slot duration, thereby meeting low latency service requirements [36]. The slot duration is 1, 0.5, 0.25, and 0.125 ms when the SCS is 15, 30, 60, and 120 kHz, respectively. The number of time slots in a subframe is 1, 2, 4 and 8. A slot is composed of several orthogonal frequency division multiplexing (OFDM) symbols, with 14 and 12 OFDM symbols when using normal cyclic prefix and extend cyclic prefix, respectively.

The resource unit in the time and frequency domains is a subframe and subcarrier, respectively. The composition of a time–frequency resource involves resource elements (REs), resource blocks (RBs), and subchannels [37]. An OFDM symbol in the time domain and a subcarrier in the frequency domain constitutes one RE. A slot and 12 consecutive subcarriers of the same SCS constitute one RB, and the bandwidth of each RB (${BW}_{RB}$) corresponds to different SCSs following ${BW}_{RB}$ equals 12 times SCS. In addition, N RBs constitute a subchannel, representing the smallest allocation unit, with N taking values of 10, 12, 15, 20, 25, 50, 75, and 100 RBs. The number of subchannels required to transmit a data packet is jointly determined by several parameters, including packet size, bandwidth, and modulation and coding scheme (MCS). Detailed illustrations and descriptions of the slot structure and physical resources can be found in [38,39].

The physical channel refers to a group of REs used for transmitting information. Specifically, each data packet (also known as a transport block, TB) has associated control data, known as sidelink control information (SCI) [40]. The TB, containing a complete data packet, occupies multiple subchannels and is transmitted via the physical sidelink shared channel (PSSCH). The SCI contains control information and undergoes a two-stage transmission for different control information in NR-V2X. The first stage of SCI, conveyed through the PSSCH, carries information such as the MCS, resources used by TB, and other resource reservation details. The second stage transmits some control information related to groupcast and unicast through the PSSCH.

### 3.2. NR-V2X Mode 2: SB-SPS

NR-V2X Mode 2 supports sidelink communication and allows users to automatically select resources [6]. Three resource selection methods exist in Mode 2: SB-SPS, sensing-based single resource selection method, and dynamic resource selection method. The latter two methods allow a UE to transmit only the current data packet with the selected resource and handle its own retransmission, rendering them suitable for aperiodic messages. In contrast, SB-SPS is designed for periodic messages, allowing continuous use of the same resource over several periods and resource reselection.

**Figure 2:**
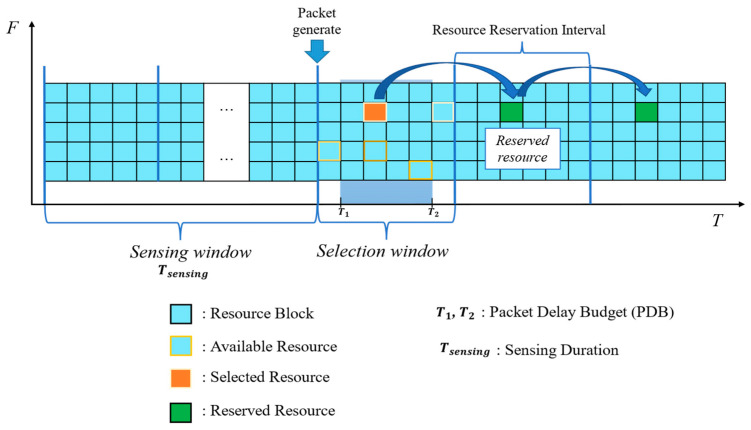
Process flow of sensing-based semi-persistent scheduling (SB-SPS).

The resource reservation interval (RRI) defines the period between resources reserved for the transmission of periodic TBs in SB-SPS. The RRI represents a time interval limited to specific values. In NR-V2X, the RRI may be {0, X, 100, 200, 300, 400, 500, 600, 700, 800, 900, 1000} ms, with X being a configurable integer within the range of [1,99]. In addition, the UE announces to its neighbors, through SCI, the resources reserved for transmitting periodic data with RRI as the period. As the focus of this work is SB-SPS, only this approach is described in detail here. The process flow of SB-SPS is shown in Figure 2, involving the following steps:

Step 1—Channel sensing: The vehicle that needs to select new resources will continuously measure the reference signal received power (RSRP) of each resource as the interference value [41]. Resources meeting one of the following two conditions are excluded. The first exclusion is related to half-duplex (HD). The resource needs to be omitted while the UE is broadcasting because it cannot receive data while it is transmitting. The second exclusion is performed when the RSRP of the resource is higher than the predefined RSRP threshold $P_{th}$. This step pertains to the sensing window. The sensing duration $T_{sensing}$ can be set as 100 ms (short-term sensing) for aperiodic transmissions or 1100 ms (long-term sensing) for periodic transmissions.

Step 2—Obtain available resource list $L_{a}$: Based on the sensing results obtained in Step 1, the list of available resources $L_{a}$ is obtained, containing resources that can be sensed by the UE with RSRP below $P_{th}$. If the number of resources in $L_{a}$ is less than X% of the total amount of resources, $P_{th}$ is increased by 3 dB, and the above steps are repeated until the number of available resources exceeds X% of all resources. The set of X% values in NR-V2X is {20%, 35%, 50%}, selected based on the configuration and service priority.

Step 3—Random selection: The UE randomly selects a resource from $L_{a}$. This stage corresponds to the selection window with two additional parameters: minimum selection time ($T_{1}$) and maximum selection time ($T_{2}$). The packet delay budget (PDB) sets the maximum allowable delay for TB transmission, which is limited by $T_{1}$ and $T_{2}$. Once the UE has selected a resource, the SPS scheme allows them to maintain the same resource for the next N consecutive transmission periods by informing neighboring devices of the reservation information via the SCI. N is also known as the reselection counter (RC). If the RRI is higher than or equal to 100 ms, the RC is generated randomly in the range [5,15] and decreased by one after each transmission.

Step 4—Resource reselection: When the RC is 0, the vehicle must select a new resource with probability (1-$P_{k}$) or retain the previous one with probability ($P_{k}$), where $P_{k}$ ϵ [0, 0.8]. Moreover, $P_{k}$ needs to be preconfigured in the simulation. In addition, two other cases require resource reselection: the new TB does not fit the previously reserved subchannel (e.g., change in TB size), or the current reservation does not satisfy the PDB of the new TB (e.g., the time between TB generation and next resource reservation is higher than the PDB requirement).

In the third step, there is no assurance that the user will choose resources with less interference, owing to random selection. Additionally, significant overlap exists in the available resource lists between adjacent users, especially in high-density scenarios, and random selection schemes can exacerbate resource conflicts. Moreover, for successful packet reception, the received SINR value must be greater than the SINR threshold. However, the final SINR is influenced not only by the distance between the transmitter and receiver but also by the interference from other users using the same resources. Details of SINR are presented in Section 5.

## 4. Proposed Scheme

In this section, we provide a detailed description of the proposed scheme. Moreover, we examine the influence of the resource selection phase in NR-V2X Mode 2 on the overall performance, focusing on an improved resource allocation method that integrates resource reuse distance judgement instead of the conventional random selection. First, the framework of the RD-RS is described, followed by the introduction of the reuse distance setting and then all considered mechanisms for resource selection.

### 4.1. Reuse Distance-Aided Resource Selection (RD-RS)

**Figure 3:**
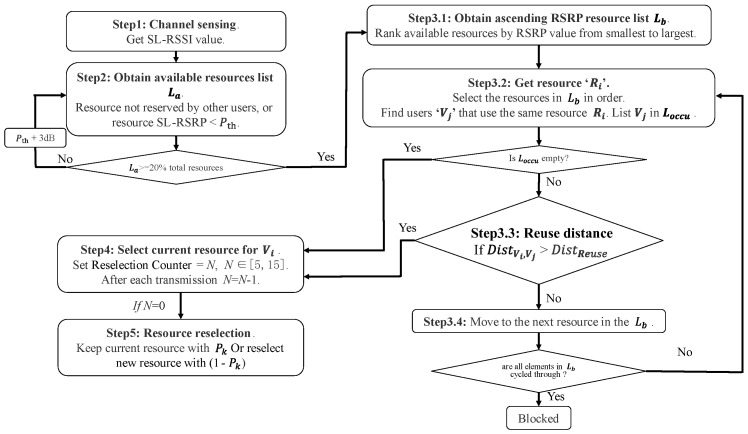
Structure of reuse distance-aided resource selection (RD-RS).

In the conventional SB-SPS scheme, vehicles randomly select resources and use them for several consecutive periods. Due to the limited resources shared among numerous users, adjacent vehicles with similar statuses result in significant overlap in the available resource lists, leading to excessive collisions and interference. In this work, the proposed method aims to improve the success ratio of packet reception and minimize resource delay resulting from random resource selection. To achieve this, the resource reuse distance judgement is incorporated into the SB-SPS algorithm. Whether a resource is selected by the current UE depends on its proximity to the neighboring UEs utilizing the same resource. This mechanism proves effective for various scenarios and does not require additional technical support, which can reduce packet loss or error reception due to resource conflicts, as well as data latency. The overall structure of the proposed scheme RD-RS is shown in Figure 3, with the left part representing conventional SB-SPS and the right part representing the reuse distance judgement scheme, and the following description pertains only to the steps in the right part.

Step 3.1—Obtain ascending-order resource list $L_{b}$: When vehicle $V_{i}$ needs to select resources, resource sensing is performed in the sensing window. Resources that are occupied or above the sensing threshold are excluded, and the available resource list $L_{a}$ is generated. Then, list $L_{b}$ is obtained by arranging each resource in $L_{a}$ in ascending order according to its RSRP value ($L_{b}$ represents $L_{a}$ ranked by RSRP in ascending order).

Step 3.2—Define resources $R_{k}$ in $L_{b}$: Resources in $L_{b}$ are selected in order, and information about other neighbors $V_{j}$ using the same current resource $R_{k}$ and corresponding distance to them is obtained. These neighbors, $V_{j}$, are treated as occupiers and included in list $L_{occu}$. Note that neighbors in $L_{occu}$ may not exist, or there may be more than one neighbor at a time.

Step 3.3—Determine the reuse distance: If $L_{occu}$ is empty, the UE $V_{i}$ can use the current resource $R_{k}$ without any further operation. However, if $L_{occu}$ is not empty, it is necessary to determine whether the distance between $V_{i}$ and the occupier $V_{j}$ (${Dist}_{V_{i},V_{j}}$) is greater than the resource reuse distance ${Dist}_{reuse}$. In the presence of multiple neighbors, it is necessary to determine each distance. Only when the distance between $V_{i}$ and all neighbors $V_{j}$ in $L_{occu}$ is greater than ${Dist}_{reuse}$ (${Dist}_{V_{i},V_{j}}$ > ${Dist}_{reuse}$), $V_{i}$ can select the current resource $R_{k}$; otherwise, we continue to judge the next resource $R_{k+1}$ in $L_{b}$.

Step 3.4—Circulate through all available resources: If a suitable resource is obtained in the previous step, this step can be omitted. Otherwise, the above steps are repeated until all resources in $L_{b}$ are cycled, and no resource with a reuse distance greater than ${Dist}_{reuse}$ is found. In this case, none of the candidate resources are suitable, the UE $V_{i}$ needs to wait for the next interval, and the current data packet is blocked.

The pseudocode for the proposed RD-RS is presented in Algorithm 1. Based on the above steps, we can predict that there are three possibilities for a UE $V_{i}$ performing resource selection via RD-RS: (1) the UE selects an available resource without any occupier; (2) the UE selects an available resource used by other occupier(s) $V_{j}$, but the distance between the current UE $V_{i}$ and $V_{j}$ is greater than the resource reuse distance, ${Dist}_{V_{i},V_{j}}$ > ${Dist}_{reuse}$; and (3) no valid resources exist, and the current data packet is blocked. RD-RS has significant utility in supporting users in selecting a resource with minimal interference from neighboring users, enhancing successful reception, and optimizing utilization of the wireless channel. This helps reduce resource selection conflicts and latency, as demonstrated in the simulation section. The concept of resource reuse distance is explained in the following contents.

### 4.2. Determination of the Reuse Distance ${Dist}_{reuse}$

- Fixed reuse distance ${Dist}_{reuse}$

In the preliminary phase of testing the RD-RS algorithm, a fixed value for reuse distance is used. This means that the same values are used for all scenarios, and the value ranges from 100 to 500 m with a step of 50 m. The objective is to assess the effectiveness of the resource selection algorithm. Moreover, based on the fundamental results, the adaptive method is developed. In the simulations, the reuse distance ${Dist}_{reuse}$ is defined as a parameter of the physical layer, which can be obtained by all vehicles during the parameter initialization stage.

- Adaptive reuse distance ${Dist}_{reuse}$

In the further stage of the design method, an adaptive reuse distance is considered. Specifically, the reuse distance is determined before resource selection, and it is adjusted according to the scenario settings. Furthermore, based on the simulation results, the optimal reuse distance value varies for each scenario, highlighting the significance of adaptive reuse distance values. And for this part, the reuse distance ${Dist}_{reuse}$ is calculated as a derived parameter of the physical layer, which can be obtained by all vehicles that in the current scenario during the parameter initialization stage.

In a highway scenario, the number of vehicles $N_{V}$ equals vehicle density ρ times road width. Thus, a higher ρ corresponds to a higher $N_{V}$. The number of resources is $N_{R}$. When assuming $N_{R}$ is fixed, a higher ρ corresponds to a lower PRR. Specifically, when $N_{R}$ is fixed, increasing $N_{V}$ will lead to increased resource competition, resulting in higher interference and packet error rates, causing a decrease in PRR. Moreover, in the case of the resource selection scheme with reuse distance judgement, a smaller ${Dist}_{reuse}$ helps achieve a superior PRR in congested scenarios, while a larger ${Dist}_{reuse}$ leads to superior performance in sparse scenarios, as illustrated in the simulation section. Therefore, the objective of this work is to enhance PRR performance by optimizing ${Dist}_{reuse}$ in different scenarios.

We define $R_{comp}$ as $N_{V}$ divided by $N_{R}$, indicating the level of resource competition in each scenario. Using the Gaussian function, $R_{comp}$ is introduced to derive the coefficient ***C**_**o**_* for determining ${Dist}_{reuse}$.

(1) $$Co=\frac{1}{\sigma \sqrt{2\pi }}e^{-\frac{(R_{comp}{-\mu )}^{2}}{2\sigma^{2}}},$$

where σ and μ are parameters of the Gaussian function, with values of 0 and 0.4, respectively. Co ensures a decreased Gaussian trend in ${Dist}_{reuse}$ as ρ increases. Moreover, because ρ is inversely proportional to $N_{R}$, a larger ρ corresponds to larger $N_{V}$, indicating more severe resource competition. Consequently, Co decreases as ρ increases. Co is substituted into Equation (2) to calculate the final ${Dist}_{reuse}$ for a specific scenario.

(2) $${Dist}_{reuse}=Min[(Co\times {Di}_{reMax}+{Di}_{reMin}),{Di}_{reMax}].$$

Here, the function Min yields the minimum value. ${Di}_{reMin}$ and ${Di}_{reMax}$ represent the minimum and maximum values of ${Dist}_{reuse}$, respectively, limiting the range of ${Dist}_{reuse}$. According to Equation (2), the following aspects are ensured: ① the value of ${Dist}_{reuse}$ is always within the allowable range of [${Dist}_{reuse\_min}$, ${Dist}_{reuse\_max}$]; ② ${Dist}_{reuse}$ decreases with increasing ρ, facilitating the optimization of its value for each scenario.

Furthermore, the values of ${Di}_{reMin}$ and ${Di}_{reMax}$ are set as Raw and ${Dist}_{max}$, respectively. The former represents the awareness range for vehicles to periodically broadcast information to neighbors within this range. The maximum distance a packet can be transmitted, known as ${Dist}_{max}$, is influenced by various parameters. The value of Raw should be smaller than ${Dist}_{max}$. The range of the reuse distance is subject to the following conditions: neither below Raw to avoid severe resource selection overlap, nor above ${Dist}_{max}$ to maintain algorithm validity. Raw is a predetermined fixed parameter in the simulation, while ${Dist}_{max}$ is derived from the following equation:

(3) $${Dist}_{max}={\left[\frac{\left(P_{tx}+G_{t}-{{BW}_{RB}}_{}\right)\times G_{r}}{{SINR}_{th}\times L_{0}\times P_{n}}\right]}^{\frac{1}{\beta }}\times 10^{\frac{2*SDLOS}{10*\beta }},$$

where $P_{tx}$ represents the transmission power; and $G_{t}$ and $G_{r}$ denote the antenna gain on the transmitter and receiver, respectively. ${{BW}_{RB}}_{}$ represents the bandwidth of an RB. The threshold SINR, denoted as ${SINR}_{th}$, determines whether a data packet is received successfully, and it is calculated using Equation (4). $L_{0}$ is a parameter for the path loss function; $P_{n}$ represents the power of noise; β is the path loss exponent of the channel model; and SDLOS is the standard deviation of shadowing in line of sight (LOS).

In this part, the value of ${Dist}_{reuse}$ depends on parameters such as the density, number of resources, *Raw*, MCS, and transmission power. Thus, ${Dist}_{reuse}$ is adaptively adjusted according to scenario settings to meet the requirements for resource reuse and enhance the overall performance.

### 4.3. Resource Selection Mechanisms

- SB-SPS

SB-SPS is a conventional resource allocation scheme in NR-V2X, as discussed in Section 3.2.

- RD-RS

RD-RS, the proposed scheme, is described in Section 4.1. The value of ${Dist}_{reuse}$ may be fixed or adaptive, as described in Section 4.2.

- Always-best resource selection

To prove the effectiveness of the proposed scheme, we consider not only the standard SB-SPS and RD-RS but also the always-best resource selection mechanism. Always-best resource selection supports the UE in selecting the best resource (i.e., the resource with the lowest RSRP) based on the ascending-order resource list (Step 3.1). The comparison of the three schemes can help verify whether the best resource with the lowest RSRP can lead to better performance than SB-SPS and highlight the importance of resource selection in NR-V2X communication.
**Algorithm 1: Pseudo-code of the proposed RD-RS method**  **Procedure reuse distance judgement of available resource**  $V_{i}$        ← Vehicles that need to select resources.  $R_{i}$        ← The resource used by the vehicle $V_{i}$.  $L_{a}$        ← Available resource list after sensing window.  $L_{b}$        ← Ascending order for RSRP values of resource in $L_{a}$.  $N_{L_{b}}$       ← Total number of resource in $L_{b}$.  k = 1       ← Resource order in $L_{b}$.  **if** k < = $N_{L_{b}}\;$    $R_{k}$      ← The k-th resource in $L_{b}$.     **if** any occupier reserved $R_{k}$         $V_{j}$      ← occupier(s).         ${Dist}_{V_{i},V_{j}}$   ← Distance between $V_{i}$ and $V_{j}$.         **if** all ${Dist}_{V_{i},V_{j}}$ > ${Dist}_{reuse\;}$         $R_{i}$ = $R_{k}$.         **else**           k = k +1           Move to line 8.          **end**     **else**
       $R_{i}$ = $R_{k}$.     **end**  **else**    $R_{i}$ = −1;       blocked.  **end**  Return $R_{i}$.

## 5. Performance Evaluation

This section describes the simulation settings and results. First, simulation assumptions are provided, including scenarios, parameter values, performance evaluation metrics, and SINR calculation details. Subsequently, the numerical simulation results are presented and analyzed.

### 5.1. Simulation Assumptions

- Scenario

Road configurations for all scenarios are detailed in Annex A of [3], with the default setting in this work being a highway scenario. A highway scenario (Figure 1) with six lanes and three lanes in each direction is considered. The length and width of the lane are 2 km and 3.5 m, respectively. The vehicle density varies according to the pre-configuration in the simulation.

For certain simulations, an urban scenario with four blocks is also considered. The total simulation area size is 866 m × 500 m, and the other settings are the same as those in [3]. The objective is to simulate an urban environment with buildings and sidewalks, with vehicles allowed to travel in both horizontal and vertical directions.

- Mobility and channel model

The vehicle UE is placed on the road according to a spatial Poisson process. The number of vehicles is determined as the product of the vehicle density and lane length on the highway. The vehicle positions are updated every 100 ms in the simulation.

The channel is ITS 5.9 GHz, and the channel model is a WINNER+ scenario, with a variance of 3 dB and decorrelation distance of 25 m [2]. The propagation type is the same as that described in [3]. The LOS configuration is used for V2V links in the highway scenario, whereas LOS and NLOS settings are adopted in the urban case.

- Parameter setting

Table 1 summarizes the meaning and adopted values of parameters regarding scenarios, physical layers, and resource allocation. A comprehensive explanation of the parameters can be found in the relevant references. Additional explanations are provided when the parameters change. Furthermore, the communication model in this work is the NR V2X sidelink communication model, and users communicate through broadcast. All UEs can transmit and receive data packets and establish neighbor lists and links based on the obtained information. Further details on the simulation model, including numerology, physical channels, and resource allocation scheme, can be found in [21]. In addition, to fairly compare the performance of the resource selection schemes, we considered the same random seeds to ensure that the traffic patterns were as similar as possible to reduce the impact of the random function on the overall performance.

### 5.2. Performance of Benchmark Algorithms

- Performance evaluation metrics

PRR [2]: Average ratio of successfully received data packets to the total number of transmissions. The PRR value is considered with the reference distance of 100 m. Additionally, we assess the error block rate, representing the ratio of the number of error packets and blocked packets to the total number of transmissions, respectively.

Range [42]: Maximum distance for PRR exceeding a set value (0.9 in this study). This parameter indicates the maximum distance at which a packet can be reliably received.

IPG [2]: Time (in seconds) between two consecutive successful receptions of a data packet at a given receiver from the same transmitter, within a given distance. IPG reflects the status of packet reception and the time required for data update.

- SINR

In V2X communication, the successful reception of a data packet is determined based on the SINR at the receiver compared with the SINR threshold. When the SINR exceeds the threshold, successful data reception is considered; otherwise, an error is considered to occur in the reception. The threshold, ${SINR}_{th}$, is calculated using Equation (4).

(4) $${SINR}_{th}=2^{\left(\frac{N_{sc}\;\times \;N_{sb}\;\times \;Q_{m}\;\times \;CR}{T_{slot}\;\times \;{BW}_{R}\;\times \;\alpha }\right)}-1,$$

where $N_{subc},\;N_{sc}$ and $N_{symbol},\;N_{sb}$ represent the number of subcarriers and symbols in an RB, respectively. $Q_{m}$ is the modulation order and CR is the effective coding rate, with values depending on the MCS setting. $T_{slot}$ is the duration of one slot. ${BW}_{RB},\;{BW}_{R}$ represents the bandwidth of each RB. α is a factor loss set as 0.4 following the recommendations in [43]. Furthermore, denoting the transmitting vehicle as i and receiving vehicle as j, the SINR at the receiver is calculated according to Equation (5).

(5) $${SINR}_{i,j}={\frac{S_{i,j}}{P_{n}+I_{k,j}}}^{},$$

(6) $$S_{i,j}=\frac{P_{tx}\times G_{r}}{L_{0}\times {d_{i,j}}^{\beta }},$$

(7) $$I_{K,j}=\sum_{k\epsilon K_{n}}^{}\left(S_{k,j}\right).$$

The numerator in Equation (5) represents the useful received power (Equation (6)), where $d_{i,j}$ is the distance between the transmitter and receiver, $P_{n}$ is the noise power, $I_{k,j}$ represents the cumulative interference, calculated using Equation (7), $K_{n}$ is a set containing all vehicles using the same RB (excluding transmitter i), and k is any vehicle in $K_{n}$.

### 5.3. Performance of Benchmark Algorithms

**Figure 4:**
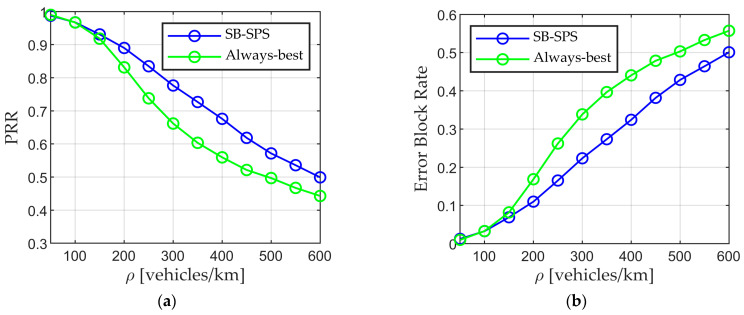
Performance of conventional SB-SPS and always-best method. (**a**) PRR vs. density and (**b**) error block rate vs. density.

The conventional SB-SPS and always-best scheme are fundamentally evaluated to demonstrate the impact of resource selection on data PRR. The results are shown in Figure 4, with the horizontal axis indicating the vehicle density ρ, and the vertical axis representing the average PRR and error block rate in (a) and (b), respectively. At low vehicle densities (i.e., 50 or 100 vehicles/km), the always-best resource approach consistently delivers a better PRR and lower error block rate than the conventional mechanism. However, the opposing results are obtained at higher densities. The reason is that the conventional SB-SPS algorithm allows users to randomly select resources from the available resources list, which not only decreases the possibility of selecting a better resource but also increases the risk of users selecting the same resource. The always-best scheme allows users to select the resource with the lowest interference. In low-density scenarios, the number of vehicles is small and sufficient resources are available, allowing each user to choose a distinct best resource, thereby improving the PRR. As the vehicle density increases, both the number of users simultaneously selecting resources and the number of neighboring users rise, resulting in severe conflicts between neighboring users regarding the best resources. Nevertheless, SB-SPS consistently outperforms the always-best scheme because random selection can mitigate this effect in high-density scenario. These results demonstrate that the resource selection algorithm plays a crucial role in data transmission. The use of appropriate algorithms can improve the quality of communication, optimize resource utilization, and minimize message update delay.

### 5.4. Performance of Fixed ${Dist}_{reuse}$–Aided RD-RS

**Figure 5:**
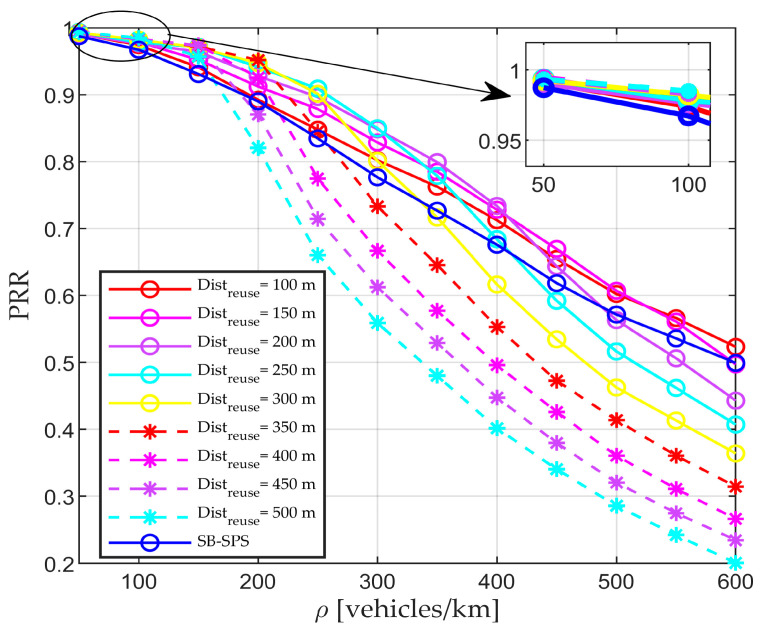
PRR performance of proposed RD-RS with fixed reuse distances.

This section comprehensively outlines the performance of the proposed RD-RS scheme. Additionally, the performances of the proposed scheme and the two benchmark methods are compared. The first simulation is designed to evaluate the performance of fixed ${Dist}_{reuse}$–aided RD-RS, and the results are presented in Figure 5. The reuse distance ${Dist}_{reuse}$ varies from 100 to 500 m, with a step size of 50 m. Clearly, the value of the reuse distance at which the maximum PRR can be achieved varies with density. In other words, no one fixed value can always guarantee the best PRR in every scenario. However, overall, as the density increases, a smaller reuse value corresponds to a superior performance. This distinction becomes particularly pronounced in high-density scenarios. The reason is that the resources are sufficient in low-density scenarios, and a larger ${Dist}_{reuse}$ can better ensure that neighboring users use different resources. In contrast, resource competition is significant in high-density scenarios. A larger ${Dist}_{reuse}$ may fail to provide legal resources, resulting in the blocking of the current packets.

**Figure 6:**
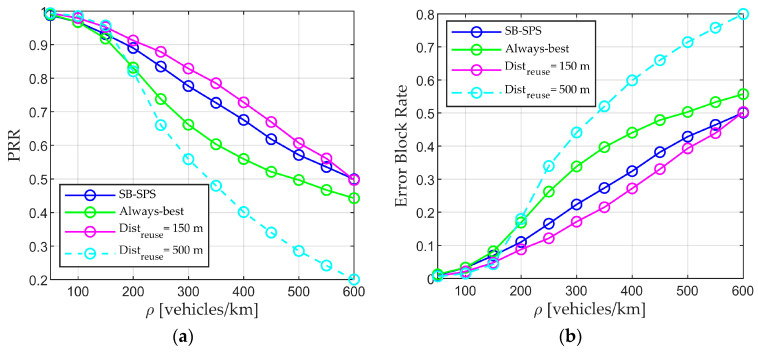
Performance of SB-SPS, always-best, and RD-RS methods with fixed reuse distance values. (**a**) PRR vs. density and (**b**) error block rate vs. density.

Additionally, the best (${Dist}_{reuse}$ of 150 m) and worst (${Dist}_{reuse}$ of 500 m) cases in Figure 5 are selected for comparison with the two benchmark algorithms, as shown in Figure 6. Although fixed ${Dist}_{reuse}$ values allow RD-RS to attain superior performance in certain scenarios, this effectiveness does not extend to all scenarios. For instance, a larger reuse distance value (dotted light blue line) should be employed at lower densities (50, 100, and 150 vehicles/km) to achieve better performance compared with the SB-SPS and always-best scheme. Conversely, at higher densities (greater than 150 vehicles/km), a smaller value (solid purple line) is necessary to achieve better results. If this value remains unchanged, the RD-RS may exhibit poor performance. Therefore, it is necessary to adaptively determine ${Dist}_{reuse}$ by considering the vehicle density and other parameters.

### 5.5. Performance of Adaptive ${Dist}_{reuse}$–Aided RD-RS

**Figure 7:**
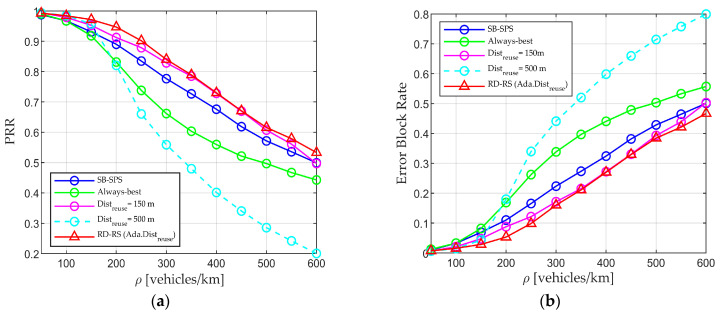
Performance of SB-SPS, always-best, RD-RS with fixed reuse distance, and RD-RS with adaptive reuse distance value. (**a**) PRR vs. density and (**b**) error block rate vs. density.

**Figure 8:**
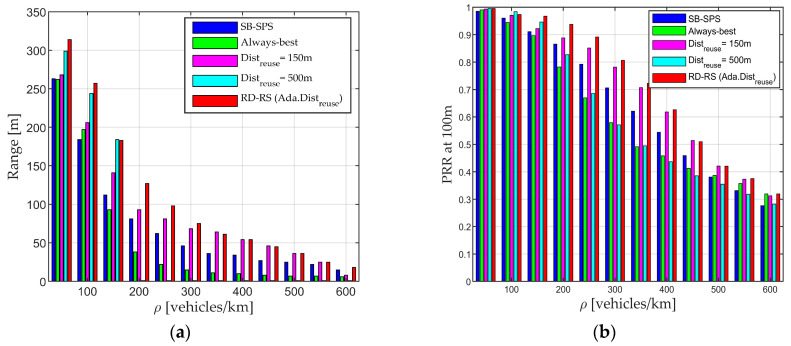
Performance of SB-SPS, always-best, RD-RS with fixed reuse distance, and RD-RS with adaptive reuse distance value. (**a**) Range and (**b**) PRR at 100 m.

Building upon the above-mentioned findings, the importance of the resource selection algorithm is validated. Specifically, we explore the performance of adaptive ${Dist}_{reuse}$–aided RD-RS. Figure 7 shows the PRR, and Figure 8 shows the Range and PRR at 100 m. ${Dist}_{reuse}$ is calculated using Equation (1) across scenarios. The minimum and maximum values allowed for ${Dist}_{reuse}$ are Raw and ${Dist}_{max}$, respectively.

The adaptive ${Dist}_{reuse}$ (solid red line) leads to higher performance in most cases compared with the fixed ${Dist}_{reuse}$ setting. The reason is that with the fixed value setting, the value may be either too large, resulting in a lack of available resources for transmission, or too small, leading to more severe conflicts in resource selection. In contrast, the adaptive setting allows for varying ${Dist}_{reuse}$ values depending on vehicle density and other parameters. The proposed approach allows UEs to select resources with a reuse mechanism to minimize interference, while alleviating the need for reuse distance in high-density situations, ensuring sufficient valid resources and reducing packet blocking rate. Figure 8 confirms the overall trend mentioned earlier, where the proposed RD-RS consistently outperforms benchmark algorithms in terms of Range and PRR at 100 m. However, the performance for the fixed ${Dist}_{reuse}$ varies depending on the scenario. For example, a ${Dist}_{reuse}$ of 500 m (light blue bars) leads to a better performance than when ${Dist}_{reuse}$ is 150 m (purple bars) in low-density scenarios, while opposite trends are observed at high densities (ρ > 200 vehicles/km). Regardless of the scenario, RD-RS with adaptive ${Dist}_{reuse}$ (red bars) always exhibits higher performance, aligning with the observations in Figure 7. The utilization of the RD-RS scheme allows UEs to select resources with less interference, especially with an adaptive ${Dist}_{reuse}$ value, ensuring low interference and a strong useful signal. This guarantees that data packets are received at the receiver with a higher SINR than other schemes, even over long distances.

**Figure 9:**
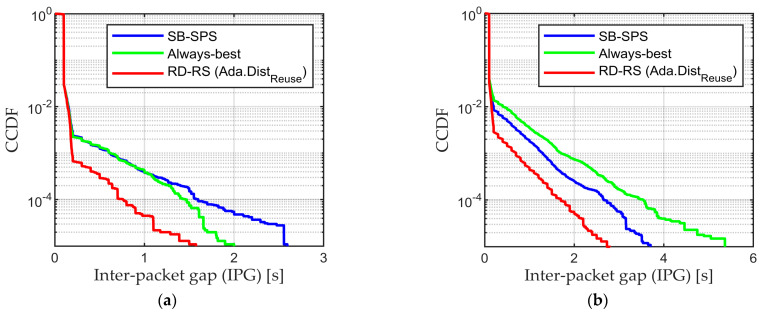
CCDF of inter−packet gap (IPG) for (**a**) ρ = 100 vehicles/km and (**b**) ρ = 200 vehicles/km.

In addition, the proposed RD-RS mechanism ensures an ample supply of valid resources, reducing the packet loss rate and significantly decreasing the IPG when compared to the conventional SB-SPS, as illustrated in Figure 9 (the red line represents RD-RS). These results show that the RD-RS algorithm consistently outperforms the conventional SB-SPS scheme, in terms of the average PRR, PRR at 100 m, Range, and delay. The adaptive ${Dist}_{reuse}$–aided RD-RS can guarantee the best performance in any scenario, thus illustrating the key role of the resource selection algorithm in this context.

**Figure 10:**
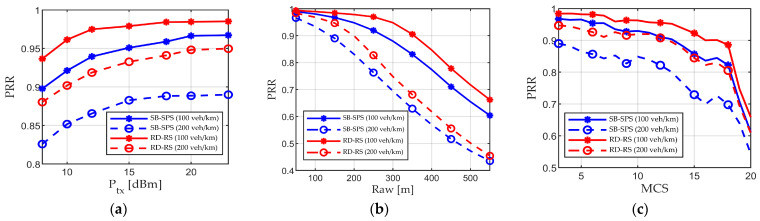
Performance of SB-SPS and adaptive ${Dist}_{reuse}$ aided RD-RS when varying (**a**) $P_{tx}$, (**b**) Raw, and (**c**) MCS.

In the preceding simulation, all parameter values remain constant, except for variations in scenario density. This part clarifies the performance of both the proposed and conventional resource allocation methods under varied parameter values, including $P_{tx}$, Raw, and MCS, as shown in Figure 10. These parameters not only influence the accuracy of data reception by determining the SINR value but also impact the reuse distance ${Dist}_{reuse}$. Two scenarios, representing sparse and congested highway cases, are considered, with ρ equal to 100 and 200 vehicles/km, respectively. The solid line represents the former scenario, while the dashed line represents the latter scenario. The blue line represents the conventional SB-SPS, while the red line represents the proposed RD-RS with adaptive ${Dist}_{reuse}$. The transmit power $P_{tx}$ is varied between 8 and 23 dBm, and other parameters remain the same as those listed in Table 1. The results are shown in Figure 10a. Clearly, the performance improves as the transmission power $P_{tx}$ increases. This is because higher transmit power ensures a higher useful signal. Second, Raw ranges between 50 and 500 m, and other parameters remain the same as those listed in Table 1. The results are shown in Figure 10b. A larger Raw corresponds to a wider range of performance evaluation, resulting in reduced PRR. In addition, the MCS is varied from 3 to 20, and other parameters remain the same as those listed in Table 1. The results are shown in Figure 10c. Overall, as the MCS increases, the PRR decreases. This is because a larger MCS corresponds to a larger ${SINR}_{th}$ [21]; thus, successful data reception requires greater useful signal power. Regardless of the setting, the proposed RD-RS can offer a higher PRR than conventional approaches. The reason is that the ${Dist}_{reuse}$ value derived from Equations (1) and (2) considers resource competition at varying densities as well as other parameter values, ensuring the effectiveness of the reuse distance and high quality of the selected resources.

**Figure 11:**
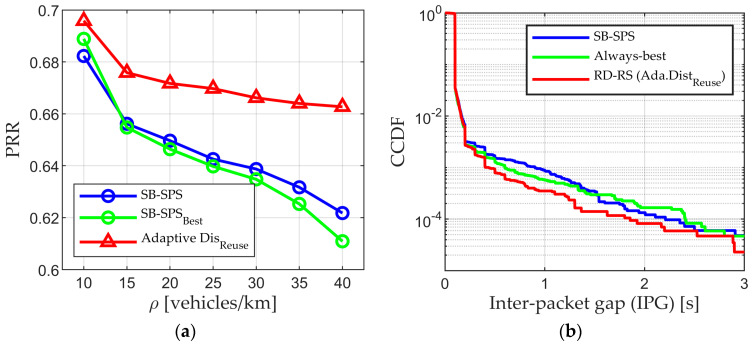
Performance in urban scenarios: (**a**) PRR; (**b**) IPG.

In addition, we assess the performance in an urban situation to confirm the effectiveness and feasibility of the proposed scheme. The results of PRR and IPG are shown in Figure 11a,b, respectively. In the urban scenario, roads are arranged both vertically and horizontally, and when the vehicle density ρ is equal to 10 and 40 vehicle/km, approximately 100 and 550 vehicles are present, respectively. Unlike highways, urban scenarios include both LOS and NLOS caused by buildings. Thus, the UEs need quality resources to receive and transmit messages. The PRR in Figure 11 is less than that in the highway scenario due to NLOS. The performance of the conventional mechanism is inferior due to the random resource selection, while the performance of the proposed scheme is superior. This is expected because the RD-RS reduces the probability that the UE selects the same resource as its neighbor and helps select resources with minimal interference and conflict. Additionally, the ${Dist}_{reuse}$ value varies based on the scene conditions, ensuring the effectiveness of the algorithm. Based on all the results and analysis, it can be concluded that regardless of the scenario, the proposed adaptive ${Dist}_{reuse}$ aided RD-RS can provide higher PRR and lower latency than the conventional scheme.

## 6. Conclusions

This paper proposes a resource selection method in NR-V2X, named reuse distance-aided resource selection (RD-RS). This method allows the reuse distance to be fixed or adaptive. The performance of a fixed reuse distance varies significantly across different scenarios, while the adaptive method ensures stable performance in any scenario. Compared with conventional SB-SPS and always-best resource selection methods, the proposed scheme can achieve superior performance. The evaluation metrics include the PRR, and error rate, Range of PRR exceeding 0.9, and PRR at a specific distance (100 m) in multiple scenarios. Furthermore, to assess the quality of the selected resource, we examine the IPG, highlighting that the RD-RS scheme can reduce delays, ensuring accurate and timely data transmission. In future work, to develop a more efficient resource selection algorithm, it will be necessary to consider the influence of other parameters. Additionally, the use of reinforcement learning can be explored to improve the alignment of reuse distance variation with scenario requirements, thereby achieving superior performance.

## Figures and Tables

**Table 1:** Parameter settings.

Parameter	Value
**Scenario (Highway)** [3]	
Lanes	Three lanes in each direction
Length	2 km
Width	3.5 m
Vehicle density (ρ)	50 to 600 vehicles/km
**Scenario (Urban)** [3]	
Lanes	Two lanes in each direction
Length	500 m
Width	3.5 m
Blocks	4
Road grid size	433 m × 250 m
Vehicle density (ρ)	10 to 40 vehicles/km
**Physical and MAC layer** [39]	
Channels	ITS bands at 5.9 GHz
Bandwidth	10 MHz
Antenna gain	3 dB
Noise figure	9 dB
Propagation model	WINNER+, Scenario B1
Transmission power $P_{tx}$	23 dBm
Awareness range RAW	150 m
MCS	3 (QPSK, ${SINR}_{th}=1.0337\;dB$)
SCS	15 kHz
Subchannel size	10 RBs
**Resource allocation** [6]	
Resource reservation interval (RRI)	100 ms
Sensing duration ($T_{sensing}$)	1100 ms
Resource sensing threshold ($P_{th}$)	−110 dBm
Available resource ratio (X%)	20%
Resource keep probability ($P_{k}$)	0.4
Min & Max time for allocation ($T_{1}$, $T_{2}$)	0 ms and 100 ms

## Data Availability

Data are contained within the article.

## References

[1] 3GPP (2022). Service Requirements for V2X Services; Stage 1; TS 22.185 V17.0.0; 3rd Generation Partnership Project. https://portal.3gpp.org/desktopmodules/Specifications/SpecificationDetails.aspx?specificationId=2989.

[2] 3GPP (2016). Study on LTE-Based V2X Services; 3GPP TR 36.885 V14.0.0; 3rd Generation Partnership Project. https://portal.3gpp.org/desktopmodules/Specifications/SpecificationDetails.aspx?specificationId=2934.

[3] 3GPP (2019). Study on Evaluation Methodology of New Vehicle-to-Everything (V2X) Use Cases for LTE and NR; 3GPP TR 37.885 V15.3.0; 3rd Generation Partnership Project. https://portal.3gpp.org/desktopmodules/Specifications/SpecificationDetails.aspx?specificationId=3209.

[4] 3GPP (2019). NR; Study on NR Vehicle-to-Everything (V2X); 3GPP TR 38.885 V16.0.0; 3rd Generation Partnership Project. https://portal.3gpp.org/desktopmodules/Specifications/SpecificationDetails.aspx?specificationId=3497.

[5] 3GPP (2022). Enhancement of 3GPP Support for V2X Scenarios; Stage 1; 3GPP TS 22.186 V17.0.0; 3rd Generation Partnership Project. https://portal.3gpp.org/desktopmodules/Specifications/SpecificationDetails.aspx?specificationId=3180.

[6] 3GPP (2022). Overall Description of Radio Access Network (RAN) Aspects for Vehicle-to-Everything (V2X) Based on LTE and NR; 3GPP TR 37.985 V17.1.1; 3rd Generation Partnership Project. https://portal.3gpp.org/desktopmodules/Specifications/SpecificationDetails.aspx?specificationId=3601.

[7] Naik G., Choudhury B., Park J.-M. (2019). IEEE 802.11bd & 5G NR V2X: Evolution of Radio Access Technologies for V2X Communications. IEEE Access.

[8] Anwar W., Franchi N., Fettweis G. Physical Layer Evaluation of V2X Communications Technologies: 5G NR-V2X, LTE-V2X, IEEE 802.11bd, and IEEE 802.11p. Proceedings of the 2019 IEEE 90th Vehicular Technology Conference (VTC2019-Fall).

[9] Saad M.M., Khan M.T.R., Shah S.H.A., Kim D. (2021). Advancements in Vehicular Communication Technologies: C-V2X and NR-V2X Comparison. IEEE Commun. Mag..

[10] Garcia M.H.C., Molina-Galan A., Boban M., Gozalvez J., Coll-Perales B., Şahin T., Kousaridas A. (2021). A Tutorial on 5G NR V2X Communications. IEEE Commun. Surv. Tutor..

[11] Abdel Hakeem S.A., Hady A.A., HyungWon K. (2020). 5G-V2X: Standardization, architecture, use cases, network-slicing, and edge-computing. Wirel. Netw..

[12] Storck C.R., Duarte-Figueiredo F. (2020). A Survey of 5G Technology Evolution, Standards, and Infrastructure Associated with Vehicle-to-Everything Communications by Internet of Vehicles. IEEE Access.

[13] Bagheri H., Noor-A-Rahim M., Liu Z., Lee H., Pesch D., Moessner K., Xiao P. (2021). 5G NR-V2X: Toward Connected and Cooperative Autonomous Driving. IEEE Commun. Stand. Mag..

[14] Lien S.-Y., Deng D.-J., Lin C.-C., Tsai H.-L., Chen T., Guo C., Cheng S.-M. (2020). 3GPP NR Sidelink Transmissions Toward 5G V2X. IEEE Access.

[15] Wei R. (2022). Performance Analysis of Semi-Persistent Scheduling throughput in 5G NR-V2X: A MAC Perspective 2022. arXiv.

[16] Shin C., Farag E., Ryu H., Zhou M., Kim Y. (2023). Vehicle-to-Everything (V2X) Evolution From 4G to 5G in 3GPP: Focusing on Resource Allocation Aspects. IEEE Access.

[17] Molina-Masegosa R., Gozalvez J., Sepulcre M. Configuration of the C-V2X Mode 4 Sidelink PC5 Interface for Vehicular Communication. Proceedings of the 2018 14th International Conference on Mobile Ad-Hoc and Sensor Networks (MSN).

[18] Gonzalez-Martín M., Sepulcre M., Molina-Masegosa R., Gozalvez J. (2019). Analytical Models of the Performance of C-V2X Mode 4 Vehicular Communications. IEEE Trans. Veh. Technol..

[19] Yin J., Hwang S.-H. (2022). Adaptive sensing-based semipersistent scheduling with channel-state-information-aided reselection probability for LTE-V2V. ICT Express.

[20] Ali Z., Lagén S., Giupponi L., Rouil R. (2021). 3GPP NR V2X Mode 2: Overview, Models and System-Level Evaluation. IEEE Access.

[21] Todisco V., Bartoletti S., Campolo C., Molinaro A., Berthet A.O., Bazzi A. (2021). Performance Analysis of Sidelink 5G-V2X Mode 2 through an Open-Source Simulator. IEEE Access.

[22] Sehla K., Nguyen T.M.T., Pujolle G., Velloso P.B. (2022). Resource Allocation Modes in C-V2X: From LTE-V2X to 5G-V2X. IEEE Internet Things J..

[23] Thanh Le T.T., Moh S. (2021). Comprehensive Survey of Radio Resource Allocation Schemes for 5G V2X Communications. IEEE Access.

[24] Molina-Masegosa R., Gozalvez J. System Level Evaluation of LTE-V2V Mode 4 Communications and Its Distributed Scheduling. Proceedings of the 2017 IEEE 85th Vehicular Technology Conference (VTC Spring).

[25] He X., Lv J., Zhao J., Hou X., Luo T. (2020). Design and Analysis of a Short-Term Sensing-Based Resource Selection Scheme for C-V2X Networks. IEEE Internet Things J..

[26] Wen X., Peng M., Zhang X., Yan S., Li Y. Enhanced Sensing-Based Resource Scheduling Algorithm for 5G V2V Communications. Proceedings of the 2019 IEEE/CIC International Conference on Communications in China (ICCC).

[27] Abbas F., Liu G., Fan P., Khan Z., Bute M.S. A Vehicle Density based Two-Stage Resource Management Scheme for 5G-V2X Networks. Proceedings of the 2020 IEEE 91st Vehicular Technology Conference (VTC2020-Spring).

[28] Yang J., Pelletier B., Champagne B. Enhanced autonomous resource selection for LTE-based V2V communication. Proceedings of the 2016 IEEE Vehicular Networking Conference (VNC).

[29] Liang L., Ye H., Yu G., Li G.Y. (2020). Deep-Learning-Based Wireless Resource Allocation with Application to Vehicular Networks. Proc. IEEE.

[30] Fan C., Li B., Wu Y., Zhang J., Yang Z., Zhao C. (2021). Fuzzy Matching Learning for Dynamic Resource Allocation in Cellular V2X Network. IEEE Trans. Veh. Technol..

[31] Suganthi E.C., Babu K.V. (2022). A two-phase fuzzy based access network selection scheme for vehicular ad hoc networks. Peer-Peer Netw. Appl..

[32] Choi J.-Y., Jo H.-S., Mun C., Yook J.-G. (2021). Deep Reinforcement Learning-Based Distributed Congestion Control in Cellular V2X Networks. IEEE Wirel. Commun. Lett..

[33] Ye H., Li G.Y. (2018). Deep Reinforcement Learning based Distributed Resource Allocation for V2V Broadcasting. Proceedings of the 2018 14th International Wireless Communications & Mobile Computing Conference (IWCMC).

[34] Ye H., Li G.Y., Juang B.-H.F. (2019). Deep Reinforcement Learning Based Resource Allocation for V2V Communications. IEEE Trans. Veh. Technol..

[35] Song Y., Xiao Y., Chen Y., Li G., Liu J. (2022). Deep Reinforcement Learning Enabled Energy-Efficient Resource Allocation in Energy Harvesting Aided V2X Communication. Proceedings of the 2022 IEEE 33rd Annual International Symposium on Personal, Indoor and Mobile Radio Communications (PIMRC).

[36] 3GPP (2022). NR; User Equipment (UE) Conformance Specification; Radio Resource Management (RRM); 3GPP TS 38.533 V17.2.0; 3rd Generation Partnership Project. https://portal.3gpp.org/desktopmodules/Specifications/SpecificationDetails.aspx?specificationId=3388.

[37] 3GPP (2021). NR; Physical Layer Procedures for Data; 3GPP TS 38.214 V16.7.0; 3rd Generation Partnership Project. https://portal.3gpp.org/desktopmodules/Specifications/SpecificationDetails.aspx?specificationId=3216.

[38] 3GPP (2023). Evolved Universal Terrestrial Radio Access (E-UTRA); Physical Channels and Modulation; TS 36.211 V17.3.0; 3rd Generation Partnership Project. https://portal.3gpp.org/desktopmodules/Specifications/SpecificationDetails.aspx?specificationId=2425.

[39] 3GPP (2021). NR; Physical Channels and Modulation; 3GPP TS 38.211 V17.0.0; 3rd Generation Partnership Project. https://portal.3gpp.org/desktopmodules/Specifications/SpecificationDetails.aspx?specificationId=3213.

[40] 3GPP (2022). Evolved Universal Terrestrial Radio Access (E-UTRA); Multiplexing and Channel Coding; 3GPP TS 36.212 V17.1.0; 3rd Generation Partnership Project. https://portal.3gpp.org/desktopmodules/Specifications/SpecificationDetails.aspx?specificationId=2426.

[41] 3GPP (2023). NR; Physical Layer Measurements; 3GPP TS 38.215 V17.3.0; 3rd Generation Partnership Project. https://portal.3gpp.org/desktopmodules/Specifications/SpecificationDetails.aspx?specificationId=3217.

[42] Bartoletti S., Masini B.M., Martinez V., Sarris I., Bazzi A. (2021). Impact of the Generation Interval on the Performance of Sidelink C-V2X Autonomous Mode. IEEE Access.

[43] 3GPP (2020). Evolved Universal Terrestrial Radio Access (E-UTRA); Radio Frequency (RF) System Scenarios; 3GPP TR 36.942 V16.0.0; 3rd Generation Partnership Project. https://portal.3gpp.org/desktopmodules/Specifications/SpecificationDetails.aspx?specificationId=2592.

